# Prognostic implications of combining EGFR‐TKIs and radiotherapy in Stage IV lung adenocarcinoma with 19‐Del or 21‐L858R mutations: A real‐world study

**DOI:** 10.1002/cam4.7208

**Published:** 2024-04-25

**Authors:** Shuai Liang, Hanyu Wang, Yingyun Zhang, Haixia Tian, Chengming Li, Dong Hua

**Affiliations:** ^1^ Department of Oncology The Affiliated Wuxi People's Hospital of Nanjing Medical University, Wuxi Medical Center Wuxi China; ^2^ Department of Radiation Oncology, Shandong Cancer Hospital and Institute Shandong First Medical University and Shandong Academy of Medical Sciences Jinan China; ^3^ The Affiliated Children's Hospital of Jiangnan University, Wuxi School of Medicine Wuxi China; ^4^ Department of oncology Shengli Oilfield Central Hospital Dongying China

**Keywords:** epidermal growth factor receptor‐tyrosine kinase inhibitor, exon 19 deletion, exon 21 L858R mutation, radiotherapy, Stage IV lung adenocarcinoma

## Abstract

**Objective:**

To elucidate the potential benefits of combining radiotherapy and epidermal growth factor receptor‐tyrosine kinase inhibitors (EGFR‐TKIs) for individuals with Stage IV lung adenocarcinoma (LUAD) harboring either exon 19 deletion (*19‐Del*) or exon 21 *L858R* mutation (*21‐L858R*).

**Methods:**

In this real‐world retrospective study, 177 individuals with Stage IV LUAD who underwent EGFR‐TKIs and radiotherapy at Shandong Cancer Hospital from June 2012 to August 2017 were included. The main focus of this real‐world study was overall survival (OS).

**Results:**

The clinical characteristics of patients with Stage IV LUAD harboring *19‐Del* were similar to those harboring *21‐L858R* (*p* > 0.05). Overall, the patients had a median OS (mOS) of 32.0 months (95% confidence interval [CI]: 28.6–35.5). Subsequently, multivariate analysis indicated that both *EGFR* mutations and thoracic radiotherapy were independent predictors of OS (*p* = 0.001 and 0.013). Furthermore, subgroup analysis highlighted a longer OS for the *19‐Del* group compared to the *21‐L858R* group, especially when EGFR‐TKIs were combined with bone metastasis or thoracic radiotherapy (mOS: 34.7 vs. 25.1 months and 51.0 vs. 29.6 months; *p* = 0.0056 and 0.0013, respectively). However, no significant differences were found in OS when considering patients who underwent brain metastasis radiotherapy (mOS: 34.7 vs. 25.1 months; *p* = 0.088).

**Conclusions:**

Patients with Stage IV LUAD harboring 19‐Del experience a notably prolonged OS following combined therapy with EGFR‐TKIs and radiotherapy, while this OS benefit is observed despite the absence of substantial differences in the clinical characteristics between the *19‐Del* and *21‐L858R* groups.

## 1 INTRODUCTION

It is widely acknowledged that lung cancer is the leading reason for cancer‐related mortality in China and globally. Approximately 80%–85% of these cases are attributed to non‐small cell lung cancer (NSCLC).^1^, ^2^ Specifically, lung adenocarcinoma (LUAD) represents approximately 60% of NSCLC cases.^3^, ^4^ Individuals with NSCLC have a relatively low 5‐year survival rate because approximately 75% of them are diagnosed when surgical intervention is no longer feasible. Recent studies have revealed an increase in the overall survival (OS) rates of individuals with advanced NSCLC who fulfill the criteria for immunotherapy, including programmed cell death 1 (PD‐1) and programmed cell death ligand‐1 (PD‐L1), or targeted therapy using epidermal growth factor receptor‐tyrosine kinase inhibitors (EGFR‐TKIs). Over 5 years, these rates have increased by approximately 15%–50%.^5^, ^6^, ^7^, ^8^, ^9^ Unfortunately, most clinical studies on immunotherapy did not include patients harboring *EGFR* mutations.

At present, targeted therapy remains a prominent treatment approach for individuals with advanced NSCLC and *EGFR* mutations, particularly for those with advanced LUAD.^3^, ^10^, ^11^ In NSCLC, approximately 85% of *EGFR* mutations are either exon 19 deletion (*19‐Del*) or exon *21 L858R* mutation (*21‐L858R*).^12^, ^13^ Brain and bone metastases encompass some of the primary contributors to treatment failure in patients with LUAD and *EGFR* mutations. These metastases often manifest during the initial diagnosis.^14^, ^15^, ^16^, ^17^ In clinical settings, individuals with advanced LUAD and *EGFR* mutations who present with symptoms of brain or bone metastasis generally receive a treatment regimen involving radiotherapy combined with EGFR‐TKIs. This approach is consistent with several studies that have revealed that the combination of EGFR‐TKIs and radiotherapy, which is applied to the primary or metastatic lesions in advanced LUAD with *EGFR* mutations, yields a remarkably longer OS duration compared with only using EGFR‐TKIs.^18^, ^19^, ^20^ Furthermore, some studies have confirmed that the Asian population has a higher frequency of *EGFR* mutations in LUAD.^4^, ^21^, ^22^ Therefore, in this real‐world retrospective study, we investigated the therapeutic effectiveness of combining EGFR‐TKIs with radiotherapy in the prognosis of individuals with Stage IV LUAD harboring either *19‐Del* or *21‐L858R* mutation.

## 2 MATERIALS AND METHODS

### 2.1 Patients

Patients diagnosed with clinical Stage IV LUAD at Shandong Cancer Hospital between June 2012 and August 2017 were included. Inclusion criteria were as follows: (I) patients aged ≥18 years with Karnofsky performance status ≥70; (II) initial diagnosis of LUAD with *19‐Del* or *21‐L858R* and confirmed diagnosis via histopathology; (III) presence of brain or/and bone metastasis, as evidenced by magnetic resonance imaging, computer tomography (CT), emission CT, x‐ray, or positron emission tomography‐CT; (IV) received EGFR‐TKIs (as first‐line or second‐line therapy) without restrictions on treatment taboos; (V) received thoracic radiotherapy for residual primary lesions, and radiotherapy for bone or/and brain metastases with clinical symptoms based on the clinicians' assessment of the potential benefits of EGFR‐TKIs; (VI) absence of a history of malignant tumors and other life‐threatening diseases; and (VII) availability of all vital clinical information. This retrospective study was approved by the Ethics Committee of Shandong Cancer Hospital. Informed consent was obtained from all individuals before receiving treatments.

### 2.2 Treatment characteristics and follow‐up

Patients received EGFR‐TKIs, including first‐generation EGFR‐TKIs (such as gefitinib, erlotinib, and icotinib) or osimertinib, as first‐ or second‐line treatment until intolerable toxicity, disease progression, or death. The radiotherapy regimen included thoracic conventional fractionation radiotherapy, whole‐brain radiotherapy (WBRT), or boost radiotherapy for local brain metastasis, and palliative radiotherapy for bone metastasis. The radiotherapy dose was 30 Gy/10 fraction or 40 Gy/20 fraction for brain metastasis, and 30 Gy/10 fraction, 24 Gy/6 fraction, or 8 Gy/1 fraction for bone metastasis. Radiation administration, target volume delineation, and organs at risk were according to the guidelines of the Radiotherapy and Oncology Group. Some patients received bevacizumab combined with chemotherapy (platinum, pemetrexed, gemcitabine, vinorelbine, docetaxel, or albumin paclitaxel) as additional systemic treatments. The first‐line chemotherapy regimens for most patients were pemetrexed plus platinum or pemetrexed plus docetaxel. Furthermore, the doses of EGFR‐TKIs, chemotherapy regimens, and bevacizumab were according to the guidelines of the Chinese Society of Clinical Oncology and the National Comprehensive Cancer Network for NSCLC. Probable treatment‐associated adverse events (AEs) were graded and analyzed using the National Cancer Institute Common Terminology Criteria for Adverse Events version 5.0. OS was the primary end point of this study. It was computed from the initiation of EGFR‐TKI therapy or radiotherapy to either death from any cause or the designated cutoff date.

### 2.3 Statistical analysis

Clinical characteristics of individuals between the *19‐Del* and *21‐L858R* groups were compared using the chi‐square test. The Cox regression model was utilized to evaluate the hazard ratio and confidence interval (CI) to investigate the effect of the clinical characteristics on OS. Survival data were evaluated using the Kaplan–Meier method. The effect of different clinical characteristics on the OS between both groups was compared using the log‐rank test. IBM SPSS Statistics 26.0 (SPSS Inc., Chicago, IL) and GraphPad Prism software version 7.0 (GraphPad Software, Inc, USA) were used to perform statistical analyses. A two‐sided *p* < 0.05 was considered statistically significant.

## 3 RESULTS

### 3.1 Patients characteristics

One hundred and seventy‐seven individuals with Stage IV LUAD who received radiotherapy combined with EGFR‐TKIs were included in this real‐world study. *19‐Del* was detected in 88 individuals (49.7%), whereas the *21‐L858R* was detected in 89 individuals (50.3%). Notably, 174 patients received first‐generation EGFR‐TKIs such as gefitinib, erlotinib, and icotinib. Among them, 17 received osimertinib after receiving first‐generation EGFR‐TKIs owing to disease progression, whereas only three received osimertinib as first‐line therapy. Table 1 summarizes the clinical characteristics of the individuals. No distinguishing traits were observed between the clinical characteristics of the *19‐Del* and *21‐L858R* groups (all *p* > 0.05).

**TABLE 1:** Baseline characteristics of all Stage IV lung adenocarcinomas between exon 19‐Del and 21‐L858R (*N* = 177).

Characteristics	Total	19‐Del	21‐L858R	*χ* ^2^	*p*‐value
		*N* = 88 (%)	*N* = 89 (%)		
Gender					
Male	57	29 (33.0)	28 (31.5)	0.045	0.832
Female	120	59 (67.0)	61 (68.5)		
Age (years)					
Median		55	52	1.013	0.314
Range		32–77	33–87		
<60	117	55 (62.5)	62 (69.7)		
≥60	60	33 (37.5)	27 (30.3)		
Smoking status					
Never	140	71 (80.7)	69 (77.5)	0.266	0.606
Former/current	37	17 (19.3)	20 (22.5)		
Brain metastasis					
No	51	30 (34.1)	21 (23.6)	2.376	0.123
Yes	126	58 (65.9)	68 (76.4)		
Bone metastasis					
No	38	19 (21.6)	19 (21.3)	0.002	0.969
Yes	139	69 (78.4)	70 (78.7)		
Brain + bone metastasis					
No	84	46 (52.3)	38 (42.7)	1.627	0.202
Yes	93	42 (47.7)	51 (57.3)		
EGFR TKIs drugs					
First generation	174	87 (98.9)	87 (97.8)	0.328	0.567
Osimertinib	3	1 (1.1)	2 (1.5)		
EGFR TKIs therapy					
First‐line	111	56 (63.6)	55 (61.8)	0.064	0.800
Second‐line	66	32 (36.4)	34 (38.2)		
Thoracic radiotherapy					
No	91	42 (47.7)	49 (55.1)	0.951	0.329
Yes	86	46 (52.3)	40 (44.9)		
Chemotherapy					
No	42	20 (22.7)	22 (24.7)	0.097	0.755
Yes	135	68 (77.3)	67 (75.3)		

Abbreviations: EGFR, epidermal growth factor receptor; TKIs, tyrosine kinase inhibitors.

### 3.2 Survival and prognostic analyses of the patients

**FIGURE 1:**
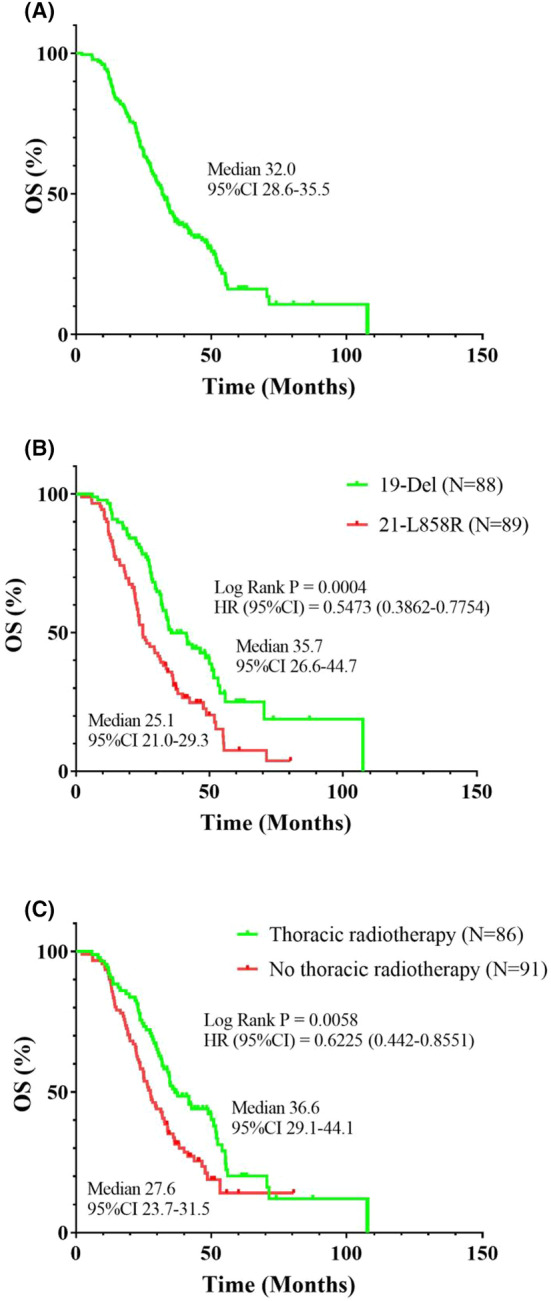
Overall survival of the all Stage IV lung adenocarcinomas patients (A) who underwent EGFR‐TKIs plus radiotherapy stratified according to EGFR mutations (B), and thoracic radiotherapy (C).

The cutoff date was April 2, 2020. And the median follow‐up duration was 45.1 (2.2–107.6) months. Till the cutoff date, 46 patients, 30 in the *19‐Del* group and 16 in the *21*–*L858R* group were still alive. All patients had a median OS (mOS) of 32.0 months (95% CI: 28.6–35.5 months) (Figure 1A). Furthermore, the 1‐, 3‐, and 5‐year OS rates of all individuals were 93.2%, 42.9%, and 27.7%, respectively. No remarkable variations were observed in the OS of the patient groups based on factors such gender, age, smoking status, presence of brain metastasis, presence of bone metastasis, EGFR‐TKIs treatment line, and chemotherapy application (all *p* > 0.05) (Figure S1A–G). Next, we used a Cox regression model to further explore the correlations between clinical characteristics and OS. In univariate analysis, we observed a significant association between *EGFR* mutations and thoracic radiotherapy and OS (*p* = 0.001 and 0.013, respectively) (Table 2). Furthermore, in multivariate analysis, both *EGFR* mutations and thoracic radiotherapy were identified as independent predictors of OS for individuals with Stage IV LUAD and either *19‐Del* or *21‐L858R* (*p* = 0.001 and 0.006, respectively). The patients in the *19‐Del* group experienced a remarkably longer OS duration than those in the *21‐L858R* group (mOS: 35.7 vs. 25.1 months; *p* = 0.0004) (Figure 1B). Moreover, the OS was significantly improved for patients with Stage IV LUAD who received thoracic radiotherapy compared with those who did not receive thoracic radiotherapy (mOS: 36.6 vs. 27.6 months; *p* = 0.0058) (Figure 1C).

**TABLE 2:** Univariable and multivariable analyses of covariable associated with OS in all Stage IV lung adenocarcinomas harboring exon 19‐Del or 21‐L858R.

Variables	Univariable analysis			Multivariable analysis		
	HR	95% CI	*p*‐value	HR	95% CI	*p*‐value
Gender (male vs. female)	0.999	0.694–1.437	0.995			
Age (years) (<60 vs. ≥60)	0.804	0.555–1.165	0.248			
Smoking status (never vs. former/current)	1.197	0.793–1.808	0.391			
Brain metastasis (no vs. yes)	1.331	0.899–1.970	0.153			
Bone metastasis (no vs. yes)	1.338	0.852–2.101	0.206			
EGFR mutations (19‐Del vs. 21‐L858R)	1.360	1.142–1.619	0.001	1.338	1.123–1.594	0.001
EGFR TKIs therapy (first‐line vs. second‐line)	0.989	0.696–1.406	0.952			
Thoracic radiotherapy (no vs. yes)	0.613	0.432–0.872	0.006	0.641	0.450–0.912	0.013
Brain metastasis Radiotherapy (no vs. yes)	1.186	0.837–1.681	0.338			
Bone metastasis radiotherapy (no vs. yes)	1.269	0.898–1.793	0.178			
Chemotherapy (no vs. yes)	0.878	0.572–1.346	0.551			

### 3.3 Subgroup analysis of individuals with LUAD in the 19‐Del and 21‐L858R groups

**FIGURE 2:**
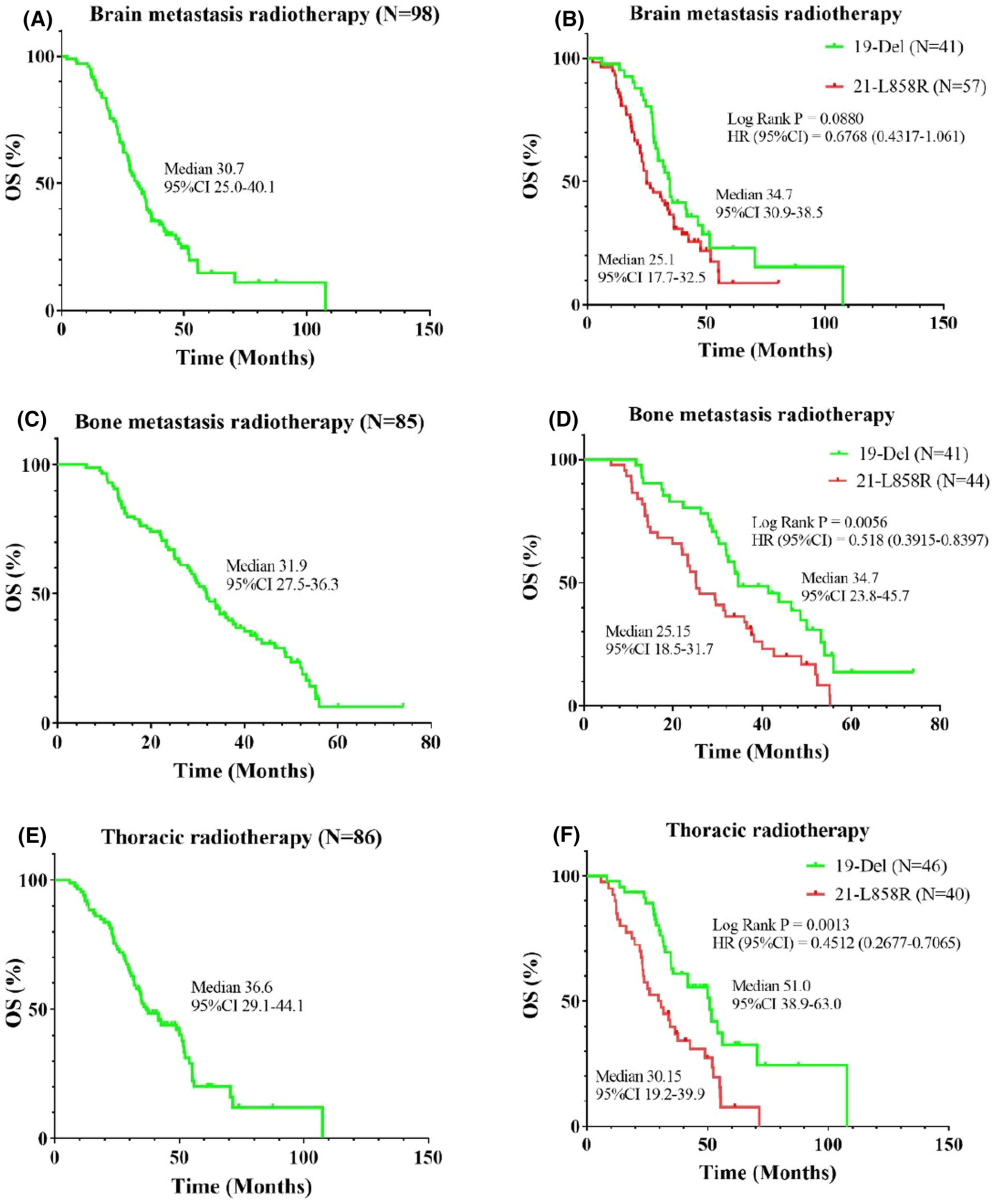
Overall survival of Stage IV lung adenocarcinomas patients harboring EGFR 19 deletion or 21 L858R underwent brain metastasis radiotherapy (A, B) or bone metastasis radiotherapy (C, D) or thoracic radiotherapy (E, F).

The differences in the prognosis and clinical characteristics of individuals with LUAD harboring *19‐Del* or *21‐L858R* still remain controversial.^5^, ^7^, ^8^, ^23^, ^24^ Therefore, we performed subgroup analysis to investigate the clinical differences between the *19‐Del* and *21‐L858R* groups as well as the differences in prognosis after receiving targeted therapy and radiotherapy. Owing to differences in clinical presentations, not all patients with LUAD underwent radiotherapy for metastatic lesions in this real‐world study. Ninety‐eight patients (98/126) with bone metastasis‐induced oppressive symptoms and pain received radiotherapy for bone metastasis, whereas 85 patients (85/139) with brain metastasis‐induced intracranial hypertension and neurological symptoms received radiotherapy for brain metastasis. Interestingly, the clinical characteristics of the patients in the *19‐Del* and *21‐L858R* groups were similar (all *p* > 0.05) (Table 3). The mOS of individuals with Stage IV LUAD who received EGFR‐TKIs combined with radiotherapy (brain metastasis, bone metastasis, or thoracic radiotherapy) was 30.7, 31.9, and 36.6 months, respectively (Figure 2A,C,E). Moreover, a comparison of the 1‐, 3‐, and 5‐year OS rates of individuals with LUAD revealed that the *19‐Del* group (96.6%, 50%, and 34.1%, respectively) significantly outperformed the *21‐L858R* group (90.0%, 34.8%, and 18.0%, respectively). The patients harboring *19‐Del* group had a significantly longer OS than those harboring *21‐L858R* (mOS: 35.7 vs. 25.1 months; 95% CI: 0.3862–0.7754; *p* = 0.0004) (Figure 1B), while no remarkable variations were observed in the OS between the 19‐Del and 21*‐L858R* groups for patients with Stage IV LUAD who received EGFR‐TKIs combined with radiotherapy for brain metastasis (mOS: 34.7 vs. 25.1 months; *p* = 0.0880) (Figure 2B). Furthermore, patients with Stage IV LUAD and *19‐Del* who received EGFR‐TKIs along with radiotherapy for bone metastasis achieved a longer OS duration than those with the *21‐L858R* mutation (mOS: 34.7 vs. 25.1 months; 95% CI: 0.3915–0.8397; *p* = 0.0056) (Figure 2D). A similar trend was observed for individuals who received EGFR‐TKIs combined with thoracic radiotherapy (mOS: 51.0 vs. 29.6 months; 95% CI, 0.2677–0.7065; *p* = 0.0013) (Figure 2F).

**TABLE 3:** Baseline characteristics of Stage IV lung adenocarcinomas underwent EGFR‐TKIs plus brain metastasis radiotherapy (*N* = 98) or bone metastasis radiotherapy (*N* = 85) between exon 19‐Del and 21‐L858R.

Characteristics	Total	19‐Del	21‐L858R	*χ* ^2^	*p*‐value	Total	19‐Del	21‐L858R	*χ* ^2^	*p*‐value
		*N* = 41 (%)	*N* = 57 (%)				*N* = 41 (%)	*N* = 44 (%)		
Gender										
Male	32	15 (36.6)	17 (29.8)	0.496	0.481	24	12 (29.3)	12 (27.3)	0.042	0.838
Female	66	26 (63.4)	40 (70.2)			61	29 (70.7)	32 (72.7)		
Age (years)										
Median		54	52	0.470	0.493		58	52	0.852	0.356
Range		32–77	33–81				32–77	34–87		
<60	73	32 (78.0)	41 (71.9)			54	24 (58.5)	30 (68.2)		
≥60	25	9 (22.0)	16 (28.1)			31	17 (41.5)	14 (31.8)		
Smoking status										
Never	76	32 (78.0)	44 (77.2)	0.010	0.920	68	32 (78.0)	36 (81.8)	0.188	0.664
Former/current	22	9 (22.0)	13 (22.8)			17	9 (22.0)	8 (18.2)		
EGFR TKIs therapy										
First‐line	54	20 (48.8)	34 (59.6)	1.139	0.286	24	31 (75.6)	30 (68.2)	0.578	0.447
Second‐line	44	21 (51.2)	23 (40.4)			61	10 (24.4)	14 (31.8)		
Thoracic radiotherapy										
Yes	45	21 (51.2)	24 (42.1)	0.798	0.372	33	15 (36.6)	18 (40.9)	0.167	0.683
No	53	20 (48.8)	33 (57.9)			52	26 (63.4)	26 (59.1)		
Chemotherapy										
Yes	73	33 (80.5)	40 (70.2)	1.335	0.248	65	32 (78.0)	33 (75.0)	0.110	0.741
No	25	8 (19.5)	17 (29.80			20	9 (22.0)	11 (25.0)		

Abbreviations: EGFR, epidermal growth factor receptor; TKIs, tyrosine kinase inhibitors.

### 3.4 Toxicity

We recorded various treatment‐associated side effects, including skin rash, radiation‐induced brain injury, radiation‐induced pneumonia, neutropenia, fatigue, anorexia, nausea, and vomiting. However, the severity of these toxicities decreased after symptomatic treatment. Importantly, we did not observe any severe (G3 or G4) toxicities or other significant acute or late toxicities.

## 4 DISCUSSION

Recently, immune checkpoint inhibitors such as PD‐1 or PD‐L1 have exhibited effectiveness against NSCLC. Notably, EGFR plays a role in immune escape by activating and enhancing PD‐L1 expression in lung cancer cells. This emphasizes the functional importance of NSCLC harboring *EGFR* mutations.^25^ However, in clinical settings, PD‐L1/1 for patients with NSCLC and *EGFR* mutations has exhibited disappointing results, as described by Gainor et al.^26^ A phase II clinical trial on PD‐L1 inhibitors revealed that the objective response rate (ORR) was 12.2% among individuals with NSCLC and *EGFR* mutations who exhibited PD‐L1 expression in at least 25% of their tumor cells. In contrast, the ORR was noted to be 3.6% for individuals with PD‐L1 expression less than 25%.^27^ Considering the limitations of immunotherapy in treating NSCLC individuals harboring *EGFR* mutations, EGFR‐TKIs remain the preferred treatment modality for individuals with this cancer type. Previous research has revealed that *EGFR* mutations, particularly *19‐Del* and *21‐L858R*, are more prominent in women, individuals with adenocarcinoma, nonsmokers, and the East Asian population. Globally, China continues to have the largest number of NSCLC patients harboring *EGFR* mutations.^4^, ^21^, ^22^ Patients with Stage IV LUAD and *EGFR* mutations substantially benefit from EGFR‐TKI therapy. However, local radiotherapy also helps alleviate symptoms and improve the quality of life.^18^, ^19^, ^20^ Therefore, we explored whether the addition of radiotherapy to EGFR‐TKI therapy can improve the survival outcomes of individuals with Stage IV LUAD carrying either *19‐Del* or *21‐L858R*. In this real‐world study, OS was noted to be strongly associated with *EFGR* mutations and thoracic radiotherapy in individuals with Stage IV LUAD carrying *EGFR* mutations. Nevertheless, no discernible variations were observed in the clinical characteristics between the individuals in the *19‐Del* and *21‐L858R* groups. In addition, subgroup analysis revealed that patients harboring *19‐Del* who received EGFR‐TKIs combined with radiotherapy (either for bone metastasis or thoracic radiotherapy) experienced a longer OS duration than those harboring *21‐L858R*. However, when considering radiotherapy for brain metastases, this distinction was not statistically significant.

In a previous study involving the American population, individuals with *19‐Del* and *21‐L858R* mutations exhibited no remarkable differences in the clinical characteristics of individuals with metastatic NSCLC.^23^ We confirmed this finding in this study and observed no differences between the two mutation groups in the Chinese population. Nevertheless, many other studies have revealed that individuals with NSCLC carrying *19‐Del* and *21‐L858R* mutations can have two separate diseases with differing underlying sensitivities to EGFR‐TKIs. In a small‐sample study involving 36 individuals with NSCLC and *EGFR* mutation who received gefitinib or erlotinib therapy, a marked dissimilarity was observed in the mOS of individuals with *19‐Del* and those with *21‐L858R* (38 vs. 17 months; *p* = 0.04).^24^ Lin et al. have reported that individuals with metastatic LUAD harboring *EGFR* mutations in exon 19 experience a better OS status after receiving EGFR‐TKIs (mOS: 33.6 vs. 23.9 vs. 27.0, month). This trend is contradictory to the findings observed for patients with *EGFR* mutations in exons 18 and 21.^5^ By combining the data from clinical trials involving individuals from LUX‐Lung 3 and LUX‐Lung 6, it was observed that individuals with Stage IIIB or IV LUAD harboring the *19‐Del* mutation and receiving afatinib therapy exhibited considerable improvements in mOS compared with those harboring the *21‐L858R* mutation (LUX‐Lung 3: 33.3 vs. 27.6 months; LUX‐Lung 6: 31.7 vs. 22.1 months; combined analysis, 31.7 vs. 22.1 months).^7^, ^8^ In this study, all individuals received both EGFR‐TKI therapy and radiotherapy. This contrasts with the findings of previous studies in which patients only received EGFR‐TKI therapy. This significant difference in treatment may play a vital role in the observed disparity between the OS of individuals with the *19‐Del* and *21‐L858R* mutations (mOS: 35.7 vs. 25.1 months; *p* = 0.0004). Interactions between radiotherapy and targeted therapy are considered intricate and include aspects such as changes in tumor radiosensitivity, oxygenation, cell cycle redistribution, and suppression of neovascularization.^28^, ^29^ Furthermore, local radiotherapy can boost tumor antigenicity (distant effect) and prevent and delay drug resistance emergence.^30^, ^31^ Many preclinical studies have also verified that EGFR‐TKIs can enhance radiosensitivity while decreasing AEs.^28^, ^32^, ^33^, ^34^ Recently, individuals with advanced LUAD and *EGFR* mutations have considerably benefited from the curative outcomes acquired using EGFR‐TKIs combined with radiotherapy for primary or metastatic lesions.^35^, ^36^ A single‐arm clinical study has suggested that individuals with advanced NSCLC and *EGFR* mutations who receive thoracic radiotherapy combined with EGFR‐TKIs can control their primary lung tumors for a prolonged duration.^37^ Yen et al. have reported that patients with unresectable Stage IIIB–IV LUAD and *EGFR* mutations who receive the combination of thoracic radiotherapy and EGFR‐TKI therapy exhibit a significantly better OS than those who only receive EGFR‐TKIs (*p* = 0.0002).^18^ Furthermore, in a study conducted by the West Japan Oncology Group 6911L, the combination of gefitinib and concurrent thoracic radiotherapy was explored in 27 patients with unresectable locally advanced NSCLC harboring *EGFR* mutations. This regimen exhibited moderate toxicity levels and achieved specific curative outcomes (mOS: 61.1 months).^19^ In addition, the SINDAS trial in China revealed that patients with synchronous oligometastatic NSCLC and *EGFR* mutations who receive EGFR‐TKIs combined with radiotherapy (thoracic or metastasis radiotherapy) can achieve a longer OS than those who only receive EGFR‐TKIs (mOS: 25.5 vs. 17.4 months; *p* < 0.01).^20^ However, our study findings suggest that thoracic radiotherapy is markedly associated with OS. In contrast, radiotherapy for brain and/or bone metastases is not significantly correlated with OS. This may be because all patients were diagnosed with Stage IV LUAD, leading to metastasis radiotherapy being administered only to those individuals with metastases and clinical symptoms.

Patients with Stage IV LUAD should receive palliative radiotherapy, possibly improving their quality of life and prolonging their survival time. Prospective nonrandomized studies have verified that radiotherapy for primary tumors (primary focus + metastatic lymph nodes in the drainage area) can improve the survival of some patients.^38^, ^39^ In a prospective study involving 26 individuals with locally advanced or metastatic NSCLC, EGFR‐TKI therapy combined with individualized thoracic radiotherapy exhibited a favorable safety profile and achieved promising outcomes (mOS: 21.8 months).^33^ In another study involving 25 Asian individuals with EGFR‐TKI‐sensitive advanced lung cancer, the early administration of thoracic radiotherapy combined with targeted therapy substantially prolonged the drug resistance onset time. This approach not only prolonged the OS (3‐year OS rate, 62.5%) but also helped effectively manage well‐tolerated toxicities.^40^ The clinical and treatment characteristics of the patients may be possible explanations for the minor OS discrepancies between the aforementioned and present studies. To maintain the long‐term control of primary lung tumors, the approach of combining thoracic radiotherapy and targeted therapy should be employed. In subgroup analysis, we observed a difference in the survival durations of individuals with the *19‐Del* mutation and those with the *21‐L858R* mutation (mOS: 51.0 vs. 30.15 months; *p* = 0.0013). This variation may be attributed to the differing sensitivities to targeted therapy and radiotherapy among the patients.

Brain metastasis is the most prevalent type of distant metastasis in individuals with NSCLC and *EGFR* mutations. When analyzing clinical data, Shin et al. noted the presence of brain metastasis in 64.7% of 314 patients with LUAD and *EGFR* mutations.^41^ The combination of EGFR‐TKIs with WBRT or stereotactic radiotherapy (SRS) can not only improve the survival of patients with Stage IV LUAD and *EGFR* mutations but also maintain good tolerance. A previous retrospective study involving 78 patients with LUAD, *EGFR* mutations, and brain metastasis revealed that the mOS of the individuals who received the combination of EGFR‐TKIs and WBRT/SRS was longer than those who only received EGFR‐TKIs (36 vs. 23 months, *p* = 0.363).^42^ Moreover, researchers have reported that individuals with NSCLC, *EGFR* mutations, and brain metastasis can experience stronger improvements after receiving both icotinib and WBRT/SRS compared with icotinib alone (31.9 vs. 27.9 months, *p* = 0.237). Notably, subgroup analysis revealed that the benefits of combination therapy were more pronounced in patients with the *19‐Del* mutation than in those with the *21‐L858R* mutation (32.7 vs. 27.4 months, *p* = 0.037).^43^ In this study, the mOS of patients was comparable with that reported in previous studies. However, subgroup analysis (34.7 vs. 25.1 months, *p* = 0.088) revealed no differences in mOS between the *19‐Del* and *21‐L858R* groups when EGFR‐TKIs were combined with radiotherapy for brain metastasis. Moreover, radiotherapy for brain metastasis was not associated with OS in all patient analyses. This may be because various EGFR‐TKIs have varying degrees of blood–brain barrier‐crossing efficacy; however, these differences were not accounted for in this study.

Aside from the brain, the bone is another common location of distant metastasis in NSCLC; this negatively affects the quality of life of people. The application of local radiotherapy is one of the most prevalent ways of avoiding and postponing AEs in the bone in individuals with lung cancer and bone metastasis.^44^ In a previous clinical study, individuals with oligometastatic NSCLC received SRS at all disease sites and the original tumor and metastatic regions and achieved satisfactory survival results.^45^ Furthermore, a retrospective study has revealed that individuals with Stage IV LUAD and *EGFR* mutations gain benefits from radiotherapy for brain or bone metastasis.^46^ Subsequently, LUAD with *EGFR* mutations responds well to thoracic radiotherapy or radiotherapy for brain/bone metastasis. Notably, individuals with the *19‐Del* mutation who received EGFR‐TKIs combined with thoracic or bone metastasis radiotherapy exhibited greater benefits compared with those with the *21‐L858R* mutation (mOS: 51.0 vs. 30.15 months, *p* = 0.0013; 34.7 vs. 25.15 months, *p* = 0.0056, respectively). One possible explanation is that complicated mutations, including *MET* mutations, tend to occur in tandem with *21‐L858R* but not with *19‐Del*; this increases the risk of poor prognosis.^47^, ^48^ Moreover, *19‐Del* is more prone to developing *T790* mutations, which are more responsive to radiotherapy than *21‐L858R* mutations.^30^, ^49^, ^50^

All patients had a mOS of 32.0 months, with tolerable toxicity, as well as 1‐, 3‐, and 5‐year OS rates of 93.2%, 42.9%, and 27.7%, respectively. These promising survival data support the hypothesis that EGFR‐TKIs combined with radiotherapy can effectively manage systemic metastases. In addition, these results emphasize the vital role of radiotherapy in delaying the progression of localized malignancies. Compared with radiotherapy for brain or bone metastasis, the combination of EGFR‐TKIs and thoracic radiotherapy not only significantly improved mOS predictability but also increased the OS period in patients (36.6 vs. 30.7 vs. 31.7 months). However, this study has several limitations, which should be addressed. First, this retrospective, single‐center study primarily focused on patients with Stage IV LUAD, making it challenging to mitigate potential selection bias. Second, we did not investigate the effects of various EGFR‐TKI types on OS. Third, research on how various treatment modalities and multisite radiotherapy affect OS outcomes is lacking. Therefore, more substantial evidence regarding the efficacy of combining radiotherapy and EGFR‐TKIs for patients with NSCLC and *EGFR* mutation could be forthcoming from various current prospective trials, including NCT02788058, NCT00973310, NCT03727867, and NCT02893332.

## 5 CONCLUSIONS

Despite the study limitations, we observed a correlation between *EGFR* mutations and thoracic radiotherapy and OS among individuals with Stage IV LUAD and *EGFR* mutations who received combination therapy with EGFR‐TKIs and radiotherapy. The differences in the clinical characteristics between the *19‐Del* and *21‐L858R* groups were insignificant. Moreover, among the individuals receiving EGFR‐TKIs combined with thoracic radiotherapy or radiotherapy for bone metastasis, those harboring the *19‐Del* mutation exhibited better OS than those harboring the *21‐L858R* mutation. In conclusion, the combination of EGFR‐TKIs and radiotherapy can serve as a safe and efficacious therapeutic approach for individuals with Stage IV LUAD and *EGFR* mutations.

## AUTHOR CONTRIBUTIONS

**Shuai Liang:** Data curation (equal); formal analysis (equal); methodology (equal); resources (equal); software (equal); validation (equal); writing – original draft (equal); writing – review and editing (equal). **Hanyu Wang:** Data curation (equal); formal analysis (equal); methodology (equal); validation (equal); writing – original draft (equal); writing – review and editing (equal). **Yingyun Zhang:** Data curation (equal); methodology (equal); validation (equal). **Haixia Tian:** Data curation (equal); software (equal); validation (equal). **Chengming Li:** Conceptualization (equal); data curation (equal); resources (equal); supervision (equal); writing – review and editing (equal). **Dong Hua:** Conceptualization (equal); data curation (equal); supervision (equal); writing – original draft (equal).

## CONFLICT OF INTEREST STATEMENT

The authors declare that they have no competing interests.

## ETHICS STATEMENT

Approval for the study protocol was granted by the Ethics Committee at Shandong Cancer Hospital. Participants willingly provided written consent to partake in the research. Additionally, permission was acquired from the individual(s) for the release of any potentially identifiable information in this publication.

## Supporting information


Figure S1.

## Data Availability

Data are available from the corresponding author upon reasonable request.
