# Supplementary material for: Prognostic implications of combining EGFR‐TKIs and radiotherapy in Stage IV lung adenocarcinoma with 19‐Del or 21‐L858R mutations: A real‐world study
Source: Cancer Med. 2024 Apr 25;13(8):e7208. doi: 10.1002/cam4.7208 (PMC11043673; doi:10.1002/cam4.7208)
Supplement: Supplementary file 1 — Figure S1. [file CAM4-13-e7208-s001.pdf]

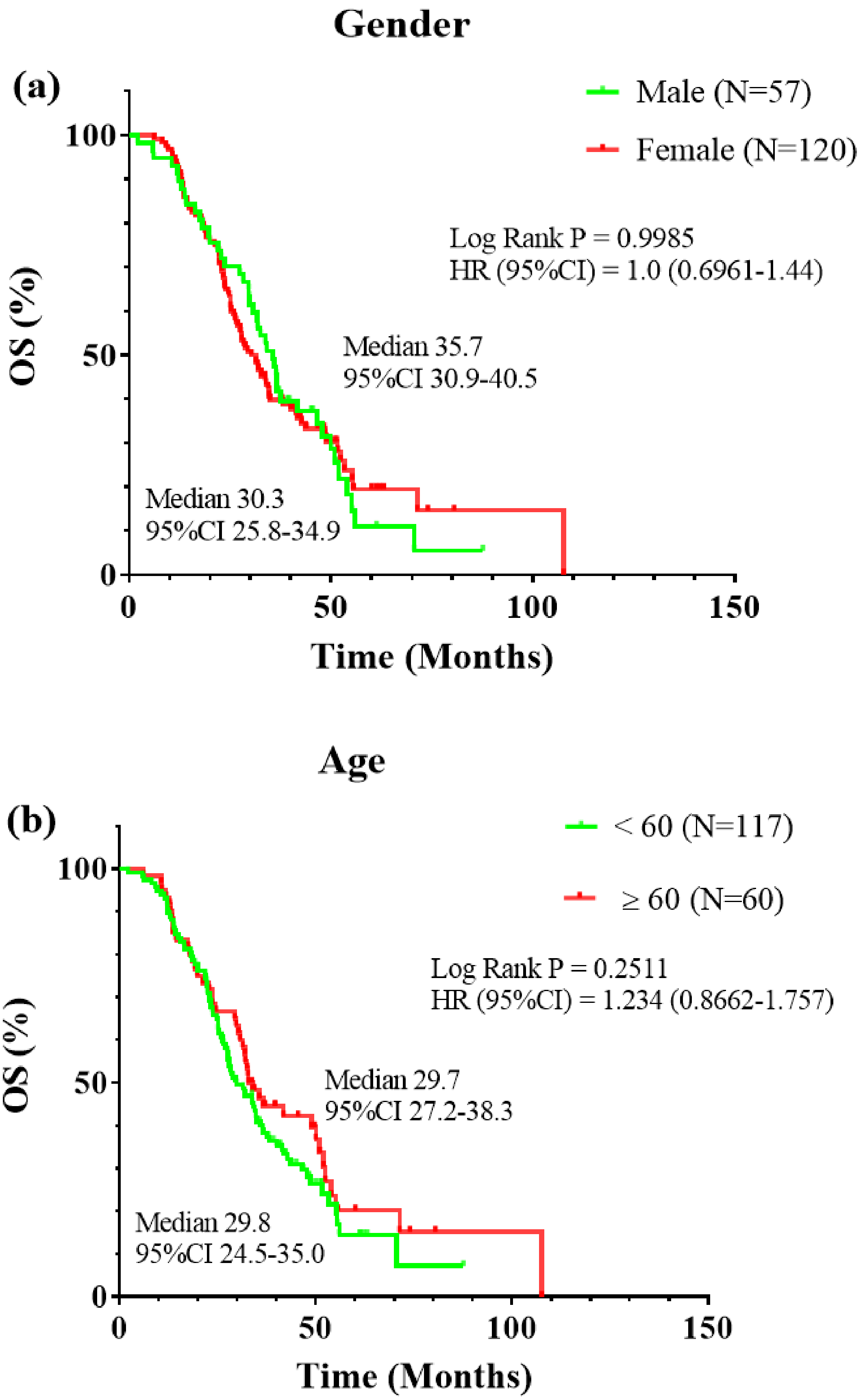
Gender
(a)
Male (N=57)
Female (N=120)
Log Rank P = 0.9985
HR (95%CI) = 1.0 (0.6961-1.44)
Median 35.7
95%CI 30.9-40.5
Median 30.3
95%CI 25.8-34.9
OS (%)
100
50
0
0
50
100
150
Time (Months)
Age
(b)
< 60 (N=117)
≥ 60 (N=60)
Log Rank P = 0.2511
HR (95%CI) = 1.234 (0.8662-1.757)
Median 29.7
95%CI 27.2-38.3
Median 29.8
95%CI 24.5-35.0
OS (%)
100
50
0
0
50
100
150
Time (Months)

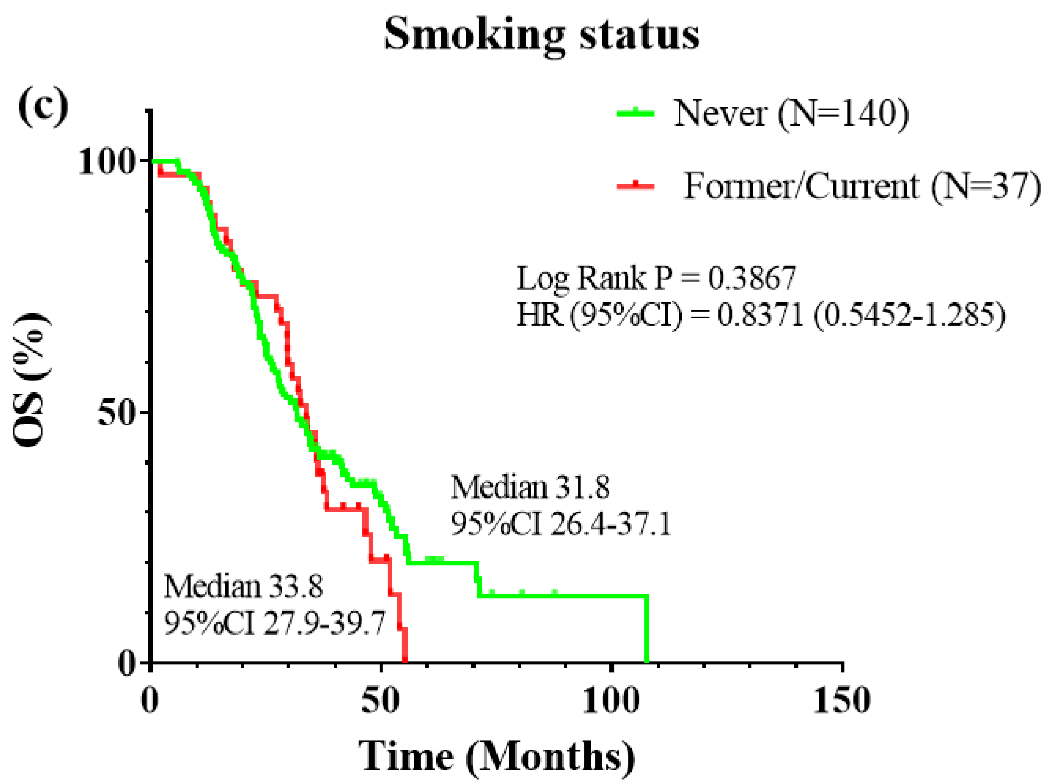
Smoking status
(c)
Never (N=140)
Former/Current (N=37)
Log Rank P = 0.3867
HR (95%CI) = 0.8371 (0.5452-1.285)
Median 31.8
95%CI 26.4-37.1
Median 33.8
95%CI 27.9-39.7
OS (%)
100
50
0
0
50
100
150
Time (Months)

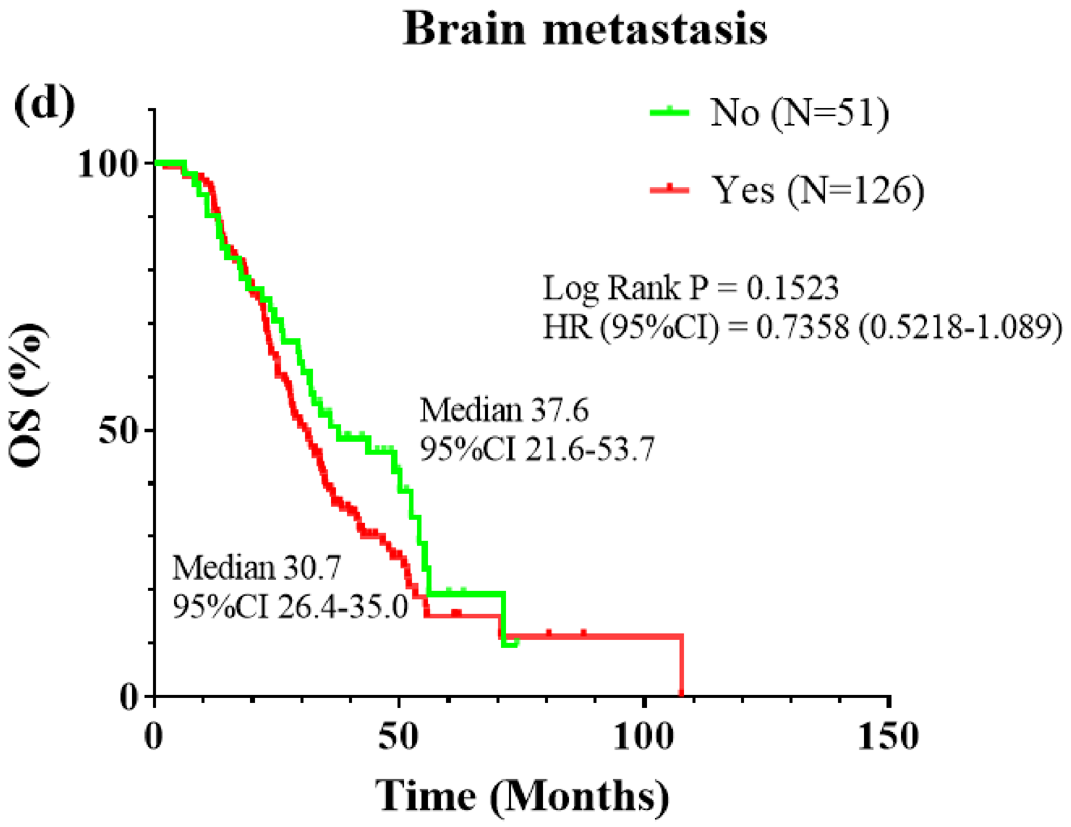
Brain metastasis
(d)
No (N=51)
Yes (N=126)
Log Rank P = 0.1523
HR (95%CI) = 0.7358 (0.5218-1.089)
Median 37.6
95%CI 21.6-53.7
Median 30.7
95%CI 26.4-35.0
OS (%)
100
50
0
0
50
100
150
Time (Months)

Bone metastasis

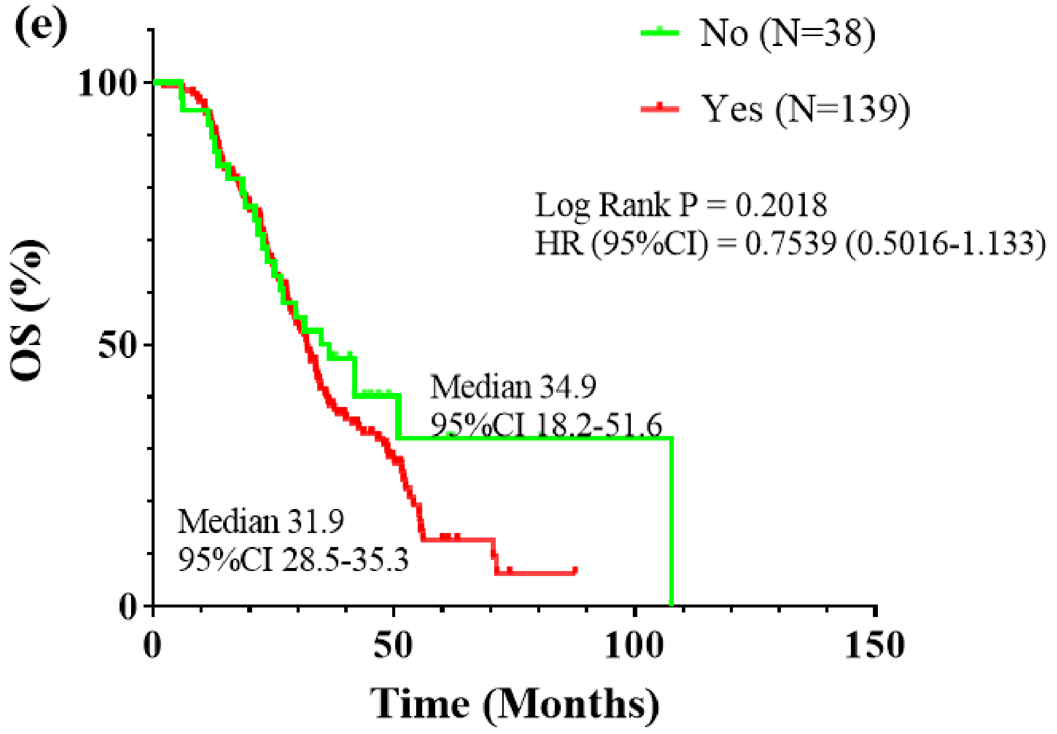

EGFR TKIs treatment

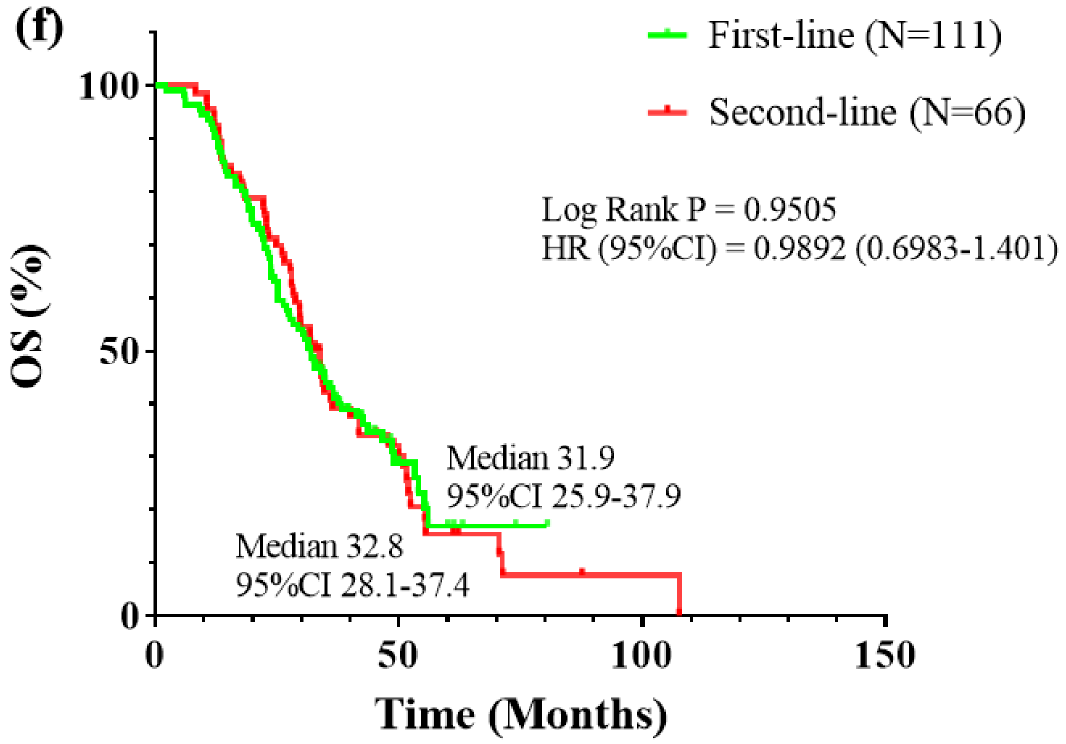

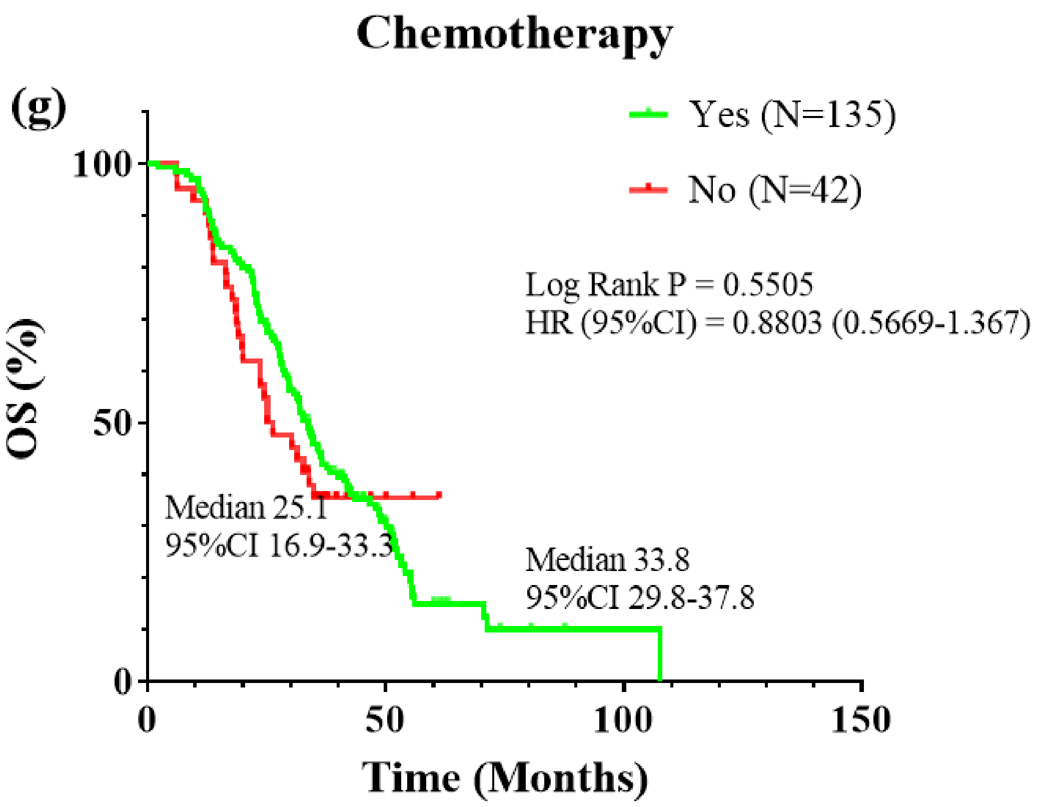

Figure S1. Overall survival of the all stage IV lung adenocarcinomas patients stratified according to gender **(a)**, age **(b)**, smoking status **(c)**, brain metastasis **(d)**, bone metastasis **(e)**, EGFR-TKIs treatment **(f)**, and chemotherapy **(g)**.
